# Supplementary material for: MicroRNA Profiling of Red Blood Cells for Lung Cancer Diagnosis
Source: Cancers (Basel). 2023 Nov 7;15(22):5312. doi: 10.3390/cancers15225312 (PMC10670279; doi:10.3390/cancers15225312)
Supplement: Supplementary file 1 [file cancers-15-05312-s001.zip › cancers-2687536-supplementary.pdf]

Supplementary files

Table S1. Among the three cell populations of cancer-free smokers, fifty miRNAs showed marked differences in expression.

miRNAs	Kruskal-Wallis statistic	P value
hsa-miR-199a-3p	58.91	0.035
hsa-miR-199a-3p	61.05	0.03
hsa-miR-199b-5p	57.94	0.019
hsa-miR-20a-5p	55.37	0.012
hsa-miR-210	58.28	0.013
hsa-miR-215	57.31	0.037
hsa-miR-221-3p	55.03	0.028
hsa-miR-223-3p	54.93	0.031
hsa-miR-23a-3p	46	0.014
hsa-miR-23b-3p	49.01	0.031
hsa-miR-25-3p	51.29	0.043
hsa-miR-27a-3p	56.97	0.033
hsa-miR-28-5p	53.09	0.028
hsa-miR-29a-3p	50.08	0.023
hsa-miR-301a-3p	57.21	0.04
hsa-miR-338-3p	62.99	0.032
hsa-miR-33a-5p	49.11	0.025
hsa-miR-342-3p	53.86	0.026
hsa-miR-363-3p	50.22	0.011
hsa-miR-365a-3p	59.98	0.04
hsa-miR-374a-5p	53.23	0.041
hsa-miR-450a-5p	65.13	0.04
hsa-miR-551b-3p	51.92	0.023
hsa-miR-582-5p	50.95	0.044
hsa-miR-766-3p	56.1	0.024
hsa-miR-92a-3p	56.34	0.017
hsa-miR-99b-5p	47.07	0.022
hsa-miR-199a-3p	58.91	0.035
hsa-miR-199a-3p	61.05	0.03
hsa-miR-199b-5p	57.94	0.019
hsa-miR-20a-5p	55.37	0.012
hsa-miR-210	58.28	0.013
hsa-miR-215	57.31	0.037
hsa-miR-221-3p	55.03	0.028
hsa-miR-223-3p	54.93	0.031
hsa-miR-23a-3p	46	0.014
hsa-miR-23b-3p	49.01	0.031
hsa-miR-25-3p	51.29	0.043
hsa-miR-27a-3p	56.97	0.033
hsa-miR-28-5p	53.09	0.028
hsa-miR-29a-3p	50.08	0.023
hsa-miR-301a-3p	57.21	0.04
hsa-miR-338-3p	62.99	0.032
hsa-miR-33a-5p	49.11	0.025
hsa-miR-342-3p	53.86	0.026

hsa-miR-363-3p	50.22	0.011
hsa-miR-365a-3p	59.98	0.04
hsa-miR-374a-5p	53.23	0.041
hsa-miR-450a-5p	65.13	0.04
hsa-miR-551b-3p	51.92	0.023

The results were analyzed by the Kruskal-Wallis test. A False Discovery Rate (FDR)-adjusted p-value of less than 0.05 was considered significant.

Table S2. Associations between the eight miRNAs and clinical and demographic data, analyzed using Pearson's correlation coefficients.

ncRNAs	Age	Sex	Race	Smoking-Pack-Years	Pulmonary Nodule Size	Histology	Tumor Stage
RBC-93-5p	-0.169	0.104	-0.172	-0.379	-0.011	-0.295	0.160
RBC-29c-3p	-0.178	-0.017	0.005*	0.158	-0.182	-0.026	-0.178
RBC-15a-5p	-0.096	0.038*	-0.299	0.073	-0.134	0.148	0.189
RBC-449b-5p	0.151	-0.231	-0.189	-0.377	0.433	-0.294	0.146
PBMC-576-3p	0.291	-0.309	-0.120	0.318	0.217	0.233	0.319
PBMC-19b-3p	0.045*	-0.048	0.034*	0.281	-0.329	0.262	-0.213
PBMC-29b-3p	-0.179	-0.216	0.050*	-0.185	-0.093	0.037*	-0.160
Neu-26a-2-3p	0.163	0.021*	0.178	-0.009	-0.126	0.015*	-0.273
Neu-574-3p	-0.251	0.008*	-0.004	0.066	0.053	0.089	0.206

* Significance at p ≤ 0.05.

Table S3. Diagnostic performances of individual miRNAs and combined miRNA panels for the detection of lung cancer, as measured by AUC analysis.

miRNAs	AUC, 95% confidence interval	P value
RBC miR-93-5p	0.7106, 0.5964 to 0.8248	0.0011
RBC miR-449b-5p	0.6151, 0.4921 to 0.7380	0.0748
RBC miR-29c-3p	0.6575, 0.5385 to 0.7766	0.0147
RBC miR-15a-5p	0.6914, 0.5746 to 0.8082	0.0030
PBMC miR-576-3p	0.6487, 0.5289 to 0.7684	0.0214
PBMC miR-19b-3p	0.6856, 0.5708 to 0.8003	0.0041
PBMC-miR-29b-3p	0.7329, 0.6244 to 0.8414	0.0003
Neutrophil miR-574-3p	0.6667, 0.5464 to 0.7869	0.0104
Neutrophil miR-26a-2-3p	0.7026, 0.5823 to 0.8229	0.0029
A panel of three RBC miRNAs (miRs-93-5p, 29C-3p, and 449-5p)	0.7552, 0.6358 to 0.8746	0.0002
A panel of three PBMC miRNAs (miR-576-3p, 19b-3p, and 29b-3p)	0.7452, 0.6327 to 0.8577	0.0003
A panel of two neutrophil miRNAs (miR-26a-2-3p and miR-574-3p)	0.6919, 0.5693 to 0.8145	0.0051
